# *ITGA5* Predicts Dual-Drug Resistance to Temozolomide and Bevacizumab in Glioma

**DOI:** 10.3389/fonc.2021.769592

**Published:** 2021-12-17

**Authors:** Ying Shi, Mengwan Wu, Yuyang Liu, Lanlin Hu, Hong Wu, Lei Xie, Zhiwei Liu, Anhua Wu, Ling Chen, Chuan Xu

**Affiliations:** ^1^ Integrative Cancer Center & Cancer Clinical Research Center, Sichuan Cancer Hospital & Institute, Sichuan Cancer Center, School of Medicine, University of Electronic Science and Technology of China, Chengdu, China; ^2^ Department of Radiation Oncology, Sichuan Cancer Hospital, Chengdu, China; ^3^ Chinese People’s Liberation Army (PLA) Institute of Neurosurgery, Chinese PLA General Hospital and PLA Medical College, Beijing, China; ^4^ The Center for Advanced Semiconductor & Integrated Micro-System, University of Electronic Science and Technology of China, Chengdu, China; ^5^ Department of Neurosurgery, The First Affiliated Hospital of China Medical University, Shenyang, China

**Keywords:** glioma, TMZ resistant, Bevacizumab resistant, *ITGA5*, vascular mimicry

## Abstract

**Aims:**

Anti-angiotherapy (Bevacizumab) is currently regarded as a promising option for glioma patients who are resistant to temozolomide (TMZ) treatment. But ongoing clinical research failed to meet therapeutic expectations. This study aimed to explore the pivotal genetic feature responsible for TMZ and Bevacizumab resistance in glioma patients.

**Methods:**

We downloaded the transcriptomic and methylation data of glioma patients from The Cancer Genome Atlas (TCGA), Chinese Glioma Genome Atlas (CGGA), and Gene Expression Omnibus (GEO) databases and grouped these patients into resistant and non-resistant groups based on their clinical profiles. Differentially expressed genes and pathways were identified and exhibited with software in R platform. A TMZ-resistant cell line was constructed for validating the expression change of the candidate gene, *ITGA5*. An *ITGA5*-overexpressing cell line was also constructed to investigate its biological function using the CCK8 assay, Western blot, periodic acid–Schiff (PAS) staining, and transcriptional sequencing.

**Results:**

Change of the cell morphology and polarity was closely associated with TMZ mono-resistance and TMZ/Bevacizumab dual resistance in glioma patients. The expression level of *ITGA5* was effective in determining drug resistance and the outcome of glioma patients, which is regulated by methylation on two distinct sites. *ITGA5* was augmented in TMZ-resistant glioma cells, while overexpressing *ITGA5* altered the cell-promoted TMZ resistance through enhancing vascular mimicry (VM) formation correspondingly.

**Conclusions:**

Both the epigenetic and transcriptional levels of *ITGA5* are effective in predicting TMZ and Bevacizumab resistance, indicating that *ITGA5* may serve as a predictor of the treatment outcomes of glioma patients.

## Introduction

Glioma is a highly aggressive primary brain tumor. The annual incidence of glioma is about 3–6/100,000 people, with a median post-diagnostic survival time of 14.6 months. The combination of radiotherapy and temozolomide (TMZ)-based chemotherapy has become the standard non-operative treatment (1). TMZ is an oral alkylating agent that methylates the guanine at *N* ^7^ and *O* ^6^ and adenine at *O* ^3^ on genomic DNA to form a mismatch during cell replication, resulting in permanent breakage of the DNA chain and cell death, consequently. However, only about 45% of glioma patients had short-term response to TMZ treatment, while the 5-year survival rate after TMZ treatment was even less than 10% in glioma patients (2). Previous research works have unearthed multiple TMZ-resistant mechanisms. For example, the *O* ^6^-methylguanine methyltransferase (MGMT)-mediated DNA repair machine can debug the TMZ-induced genomic mismatch, while epigenetic silencing of MGMT restores sensitivity to TMZ, along with longer overall survival (OS) in patients showing MGMT methylation (3, 4). Hitherto, there is no specific remedy for this complicated problem.

By means of multi-omics technology, a hypoxia environment has been proven to take charge of triggering TMZ resistance somehow. It is acknowledged that hypoxia is a key characteristic of glioma. Multiple hypoxia-responsive molecules augmented in glioma are responsible for its invasive and aggressive properties, especially hypoxia-induced factor (HIF)-1α (5). Treatment with TMZ stressed the hypoxia status by activating HIF-1α and its multiple target genes (6). The PI3K/AKT-dependent epithelial–mesenchymal transition (EMT) process was found to be activated by TMZ, which promoted malignancy as feedback (7). Additionally, stimulation of HIF-1α can also lead to the acquisition of stem status, denoted by an increased CD133 expression (8). The hypoxia-driven stem-like cell was found to express a high level of MGMT and display stronger chemotherapy resistance in turn (9). If placed under a hyperoxic condition, chemoresistant human glioma cells can be re-sensitized to TMZ (10).

Hypoxia also contributes to multidrug resistance (MDR) in a number of TMZ-resistant glioma patients. Glioma patients who relapsed with TMZ resistance feature faster tumor growth and more abundant tumor vessels. Therefore, anti-angiotherapy was proposed as an ideal option to combat the drug limitation (11). Bevacizumab is a recombinant monoclonal antibody (bevacizumab) approved for the treatment of recurrent glioma, targeting and blocking vascular endothelial growth factor (VEGF), which is a pivotal stimulator of tumor vascularization. However, several clinical trials and laboratory research have proven that current anti-VEGF anti-angiotherapy was poorly effective in TMZ-resistant glioma patients (12). The TMZ–Bevacizumab combined therapy failed to inhibit the tumor or prolong survival in glioma patients, as expected, while the molecular mechanism remains to be explored (13).

In recent years, the phenomenon of vascular mimicry (VM) has been commonly observed and considered to contribute to drug resistance (14, 15). In general, HIF-1α stimulates VEGF to boost the differentiation of pericarcinoma endothelial cells into vascular cells, namely, CD31^+^ vessels (16). Under hypoxia stress, the tumor cells themselves can differentiate into tumor-derived endothelial cells (TDECs) and form luminal structures through self-deformation and remodeling of the extracellular matrix (ECM) (17). CD31^−^ and positive periodic acid–Schiff (PAS) staining on channels of VM make it different from endothelial cell-derived vessels (18). Red blood cells can be seen in the lumen, keeping oxygen or energy supply, especially when endothelial original vessels have been damaged (19). Certainly, glioma harboring more VM presents with more malignancy and stronger invasive abilities and drug tolerance (17). The existence of VM may be responsible for dysfunction of classical anti-VEGF therapy in cutting off the VM-based oxygen and nutrition supply, thus being inefficient to eliminate the tumor (20, 21). Indeed, glioblastoma (GBM) patients with positive VM possess shorter survival times than VM-negative patients, even in patients with MGMT promoter methylation (22). Targeted intervention of tumor cell-derived blood vessels, such as the use of ibrutinib, significantly increased the response rate to chemotherapy in patients with neuroblastoma (23).

The key events of VM formation include ECM remodeling and the connection and translocation between the VM structure and tumor microvessels. These cellular activities were highly involved in the change of cell polarity and EMT, accompanied by morphological changes such as the accelerated formation of invadopodia (24). Maniotis et al. found that VM channels are rich in integrins, laminin, collagen, and heparan sulfate proteoglycan (25). Integrins are a class of cellular adhesion proteins with signal transduction function, which are widespread on the surface of tumor and neovascular cells. So far, the 18α and 9β integrin subunits have been identified to dimerize into more than 20 types, such as αVβ3 and α5β1. Integrin-α5 (*ITGA5*) was reported to impact the invasive nature of many solid tumors by promoting the EMT pathway (26). *ITGA5* located on circulating angiogenic cells have been verified to participate in neovascularization, pointing to poorer outcomes in GBM patients (27). In this study, we analyzed public data to prove the value of *ITGA5* in predicting responses to TMZ and Bevacizumab and investigated the role of *ITGA5* in mediating VM formation in glioma.

## Materials and Methods

### Public Data Acquisition

The omics data and clinical information of low-grade glioma (LGG) and GBM samples were downloaded from the Cancer Genome Atlas (TCGA; https://portal.gdc.cancer.gov/) and the Chinese Glioma Genome Atlas (CGGA; http://www.cgga.org.cn/) databases, while those of healthy samples were downloaded from the Genotype-Tissue Expression (GTEx) portal (https://www.gtexportalL.org/) (28). Six hundred and forty-eight (515 LGGs and 133 GBMs) patients with both RNA sequencing and DNA methylation data in TCGA datasets were collected and analyzed. Data of glioma patients showing TMZ/Bevacizumab dual resistance were downloaded from the Gene Expression Omnibus (GEO) database (https://www.ncbi.nlm.nih.gov/geo/; GSE79671) (29). Glioma tissue slides with ITGA5 staining were downloaded from The Human Protein Atlas (THPA) database (https://www.proteinatlas.org/). The immunostained level for each slide was evaluated using ImageJ software and compared.

### Omics Data Analysis

For transcriptional sequencing data, differentially expressed genes (DEGs) were analyzed using these data in R platform by using the “DESeq2” and “ggplot2” packages, with the definition of fold change >1.5 and false discovery rate (FDR) <0.05. Gene Ontology (GO) analysis was then presented using the DEGs, and the differentially expressed pathways or symptoms were illustrated using gene set enrichment analysis (GSEA) software. The R package “corrplot” was utilized to calculate and exhibit the correlation coefficients among the target genes. The medication and clinical follow-up information of patients were integrated to perform Kaplan–Meier analysis using the R package “survival”. The weighted correlation network analysis (WGCNA) was performed using the transcriptome data from TCGA database as the data source (30). The correlations of DEGs were systematically analyzed to calculate the parameter *β* and detect modules. Then, the relationship between the modules and TMZ resistance characteristics was investigated to determine the top-ranked modules with the strongest connections.

### Cell Lines and Cell Culture

U87MG, the human glioma cell line, was cultured in Dulbecco’s modified Eagle’s medium (DMEM) supplemented with 10% fetal bovine serum at 37°C under a humidified atmosphere of 5% CO_2_.

For the *ITGA5*-overexpressing cell line, the coding sequence of *ITGA5* (NM_002205.5) was cloned in segments and multi-fragment recombined with the PLVX-puro vector (632164; Clontech, Shiga, Japan). After confirmation of the sequence, the *ITGA5*-overexpression plasmid was transfected into 293T packaging cells with pSPAX2 and pMD2.G to produce *ITGA5*-overexpressing lentiviral particles. U87MG cells pre-seeded in six-well plates were infected using a medium containing *ITGA5*-overexpressing lentiviral particles supplemented with 10 μg/ml polybrene. At 72 h after infection, 2 μg/ml puromycin was used to screen cells overexpressing *ITGA5*, named U87MG-ITGA5.

For the TMZ-resistant GBM cell line, U87MG cells were exposed to the IC_50_ of TMZ (HY-17364; MedChemExpress, Monmouth Junction, NJ, USA) and then treated continuously with the IC_50_ of TMZ for 3 months. The TMZ-resistant subclones (U87MG-R) were isolated and maintained in DMEM with a low dose (100 μM) of TMZ.

### Cytotoxicity Assay

Cytotoxicity was measured by the sulforhodamine B (SRB) assay (Sigma-Aldrich, St. Louis, MO, USA). The cells were seeded in 96-well plates (5,000 cells/well) and cultured in the absence or presence of TMZ (250, 500, 1,000, 2,000, and 3,000 μM) for 96 h. The cell density was determined by absorbance (optical density, OD) at 490 nm. The percentages of viable cells relative to the controls (cells without previous TMZ treatment) were calculated and plotted. The IC_50_ values were calculated by derivation of the best-fit line.

### Western Blot

The total protein lysates (30 μg) of cells were separated by 10% SDS-PAGE using electrophoresis and transferred into a PVDF membrane (0.22 µm; Millipore, Bedford, MA, USA). After blocking, the membrane was probed with primary antibodies [ITGA5: 10569-1-AP (31); β-actin: 66009-1-Ig (32)] (both from Proteintech, Wuhan, China) at 1:1,000 dilution for 1 h at 37°C and then washed. Horseradish peroxidase (HRP)-conjugated anti-mouse or anti-rabbit secondary antibodies (SA00001-1 and SA00001-2; Proteintech, Wuhan, China) at 1:5,000 dilution were then used for incubation for 1 h. Immunoreactivity signals were amplified by the ECL Plus Western blotting detection system.

### PAS Staining

Cells seeded on slides were fixed with 10% formalin at room temperature for 15 min and then washed twice with phosphate-buffered saline (PBS) for PAS staining following the procedure described in *Instruction* (G1280; Solarbio, Beijing, China). The slides were treated with periodic acid solution for 10 min and then with Schiff reagent and placed in the dark for 20 min, followed by staining with hematoxylin solution for 1–2 min. Acidic differentiation solutions were added to remove excess background staining. The slides were then dehydrated, cleared, and the images collected using a microscope (Olympus, Tokyo, Japan) and the FCSnap software.

### Transcriptional Sequencing of Cells

Cells were harvested and total RNA was extracted using the Trizol reagent. Beads with oligo(dT) were used to isolate poly(A)-containing mRNA and ncRNAs after total RNA was collected. Sequenced reads were trimmed for adaptor sequence and masked for low-complexity or low-quality sequences, then mapped to the hg19 whole genome using HISAT2. The sequencing experiment was performed by HaploX Genomic Center, and the raw data were deposited in the GEO database (https://www.ncbi.nlm.nih.gov/geo/; PRJNA753670).

### Statistical Analysis

All data were analyzed using IBM SPSS Statistics for Windows software (ver. 23.0; IBM Corp., Armonk, NY, USA). To compare the EMT factor expression levels and WHO tumor grades, we employed the *χ* ^2^ test or Fisher’s exact probability test. The effects of single variables on OS or progression-free survival (PFS) were estimated by univariate analysis. Data are presented as the mean ± standard deviation (SD), unless otherwise indicated. At least three independent experiments were performed. Unless stated otherwise, the *t*-test was used to compare groups. GraphPad Prism for Windows software (ver. 6.00; GraphPad, La Jolla, CA, USA) was employed to analyze *in vitro* data, presented as the mean ± standard error of the mean (SEM). A *p*-value <0.05 was considered to reflect statistical significance.

## Results

**Figure 1:**
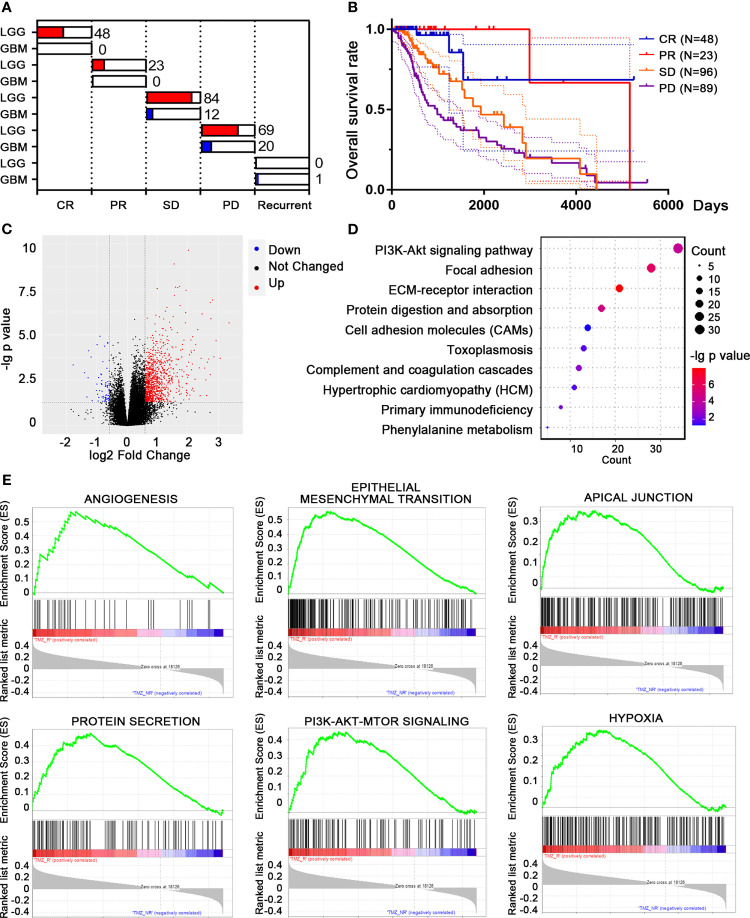
Differentially expressed genes (DEGs) and differential pathway screening between temozolomide-resistant (TMZ-R) and non-resistant (TMZ-NR) glioma patients in The Cancer Genome Atlas (TCGA) database. **(A)** Based on their responses to TMZ treatment, glioma patients from TCGA database were grouped into TMZ-R [complete response (CR), partial response (PR), stable disease (SD)] and TMZ-NR [progressive disease (PD), and recurrent] groups. **(B)** The Kaplan–Meier method was used to compute the overall survival of glioma patients with distinct response to TMZ treatment. **(C)** Volcano plot showing the DEGs between TMZ-R and TMZ-NR glioma patients. The *red points* refer to upregulated genes, while the *blue points* refer to downregulated genes in TMZ-R patients. **(D)** Pathway enrichment of DEGs by Gene Ontology (GO) analysis. **(E)** The top pathways associated with TMZ-R by gene set enrichment analysis (GSEA).

We downloaded the transcriptomic data and clinical information of LGG and GBM patients from TCGA database. A total of 167 patients who received TMZ treatment were included, classified into complete response (CR), partial response (PR), stable disease (SD), progressive disease (PD), and recurrent groups according to their disease progression and clinicopathological characteristics after TMZ treatment (**Figure 1A**). The survival rates varied among each population, while patients with PD showed the poorest outcome, as expected (**Figure 1B**). Patients with PD and recurrent outcome were classified as TMZ-resistant (TMZ-R, *n* = 59), while patients with CR, PR, and SD were classified as TMZ-non-resistant (TMZ-NR, *n* = 108). By analyzing the transcriptomic files, 974 DEGs with |fold change| > 1.5 and FDR < 0.05 were screened, including 908 upregulated and 66 downregulated genes in the TMZ-R group compared to the TMZ-NR group (**Figure 1C**). We performed GO analysis using these DEGs and found that biological processes related to cell adhesion and ECM were highly enriched, indicating that the cell morphology was greatly modified (**Figure 1D**). GSEA was also performed, displaying that clusters of EMT, PI3K/AKT pathway, and angiogenesis process were enriched in the TMZ-R group (**Figure 1E**).

**Figure 2:**
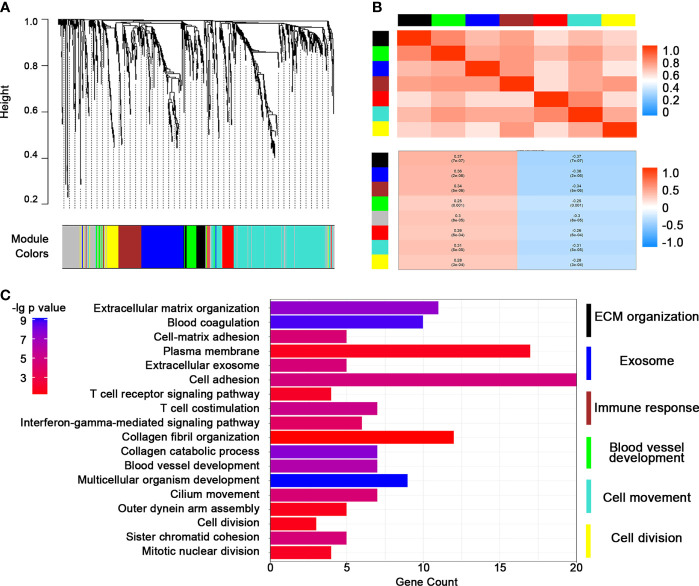
Construction of a weighted co-expression gene network by weighted correlation network analysis (WGCNA). **(A)** WGCNA dendrogram indicating the expressions of different gene modules in the above glioma samples. **(B)** Correlations between the gene modules. **(C)** Heatmap of representative pathways in each gene module.

We presented these DEGs into WGCNA streamline to construct a co-expression genetic network. Seven efficient gene modules were obtained through a one-step network construction method (**Figure 2A**). Except for the red module, the expressions of the other modules were highly positively correlated with each other, indicating a possible synergy among modules related to TMZ resistance (**Figure 2B**). The genes of each module, excluding the red module, were extracted for gene function analysis. The enrichment results of the other six modules included the following: ECM components, exosomes, immune response, vascular construction, cell movement, and cell division (**Figure 2C**). The above results supported the existence of a close association between TMZ resistance and remodeling of cell morphology, as well as the vascular status in glioma.

**Figure 3:**
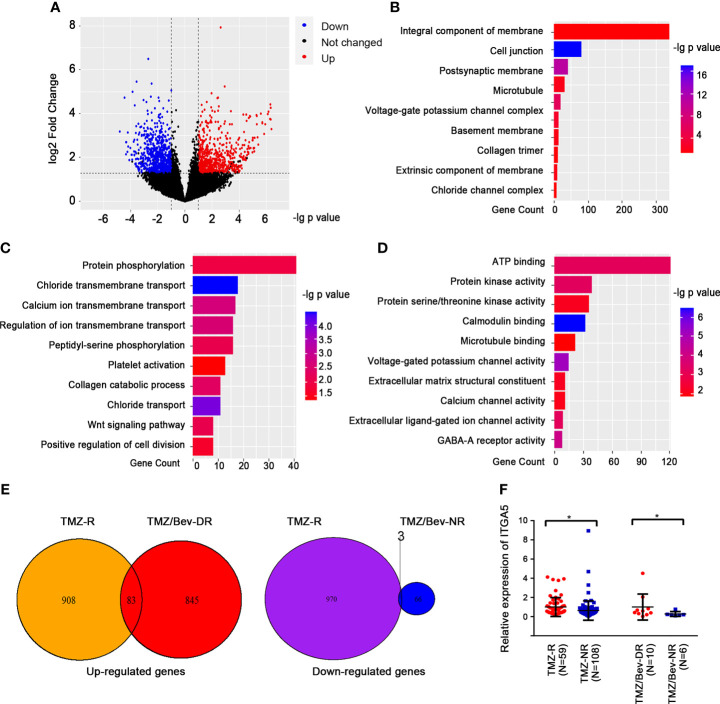
Analysis of candidate genes and related pathways in glioma patients showing dual resistance to temozolomide (TMZ) and Bevacizumab. **(A)** Volcano plots showing the differentially expressed genes (DEGs) between patients showing dual resistance to TMZ and Bevacizumab (TMZ/Bev-DR) and those not resistant to both drugs (TMZ/Bev-NR). **(B–D)** Results of the gene function cluster analysis of differential genes, including cellular components **(B)**, biological pathways **(C)**, and molecular functions **(D)**. **(E)** TMZ/Bev-DR-related differential genes were intersected with the aforementioned TMZ-R-related differential genes according to the changing trend. **(F)** Comparison of the levels of *ITGA5* in glioma patients with different responses to TMZ (and Bevacizumab) treatment (*p < 0.05).

The above results guided us to explore the potential correlation between TMZ resistance and Bevacizumab resistance. Consequently, we introduced another batch of transcriptome data from the GEO database (GSE79671). The glioma patients in this content were grouped into dual resistance (TMZ/Bev-DR) and non-resistance (TMZ/Bev-NR). A total of 845 upregulated genes and 970 downregulated genes with |fold change| > 1.5 and *p* < 0.05 were identified in the TMZ/Bev-DR group (**Figure 3A**). Following GO analysis, we found that the DEGs were responsible for coding membrane proteins, especially centering on transshipment of cellular components, collagen metabolic pathways, among others (**Figures 3B**–**D**). The two batches of DEGs from the above two datasets were intersected to obtain the overlapping factors; 83 upregulated genes and 3 downregulated genes were screened (**Figure 3E**). A broad range of genes related to cell skeleton and structure were included in these upregulated genes, such as *ITGA5*, *ICAM1*, and families of annexin (*ANXA1* and *ANXA2*) and collagen (*COL4A1*, *COL5A1*, and *COL14A1*). Considering the expression levels and biological function of these genes, *ITGA5*, which was both upregulated in the TMZ single-drug resistance and TMZ/Bevacizumab dual-drug resistance groups, was selected for further analysis and functional verification (**Figure 3F**).

**Figure 4:**
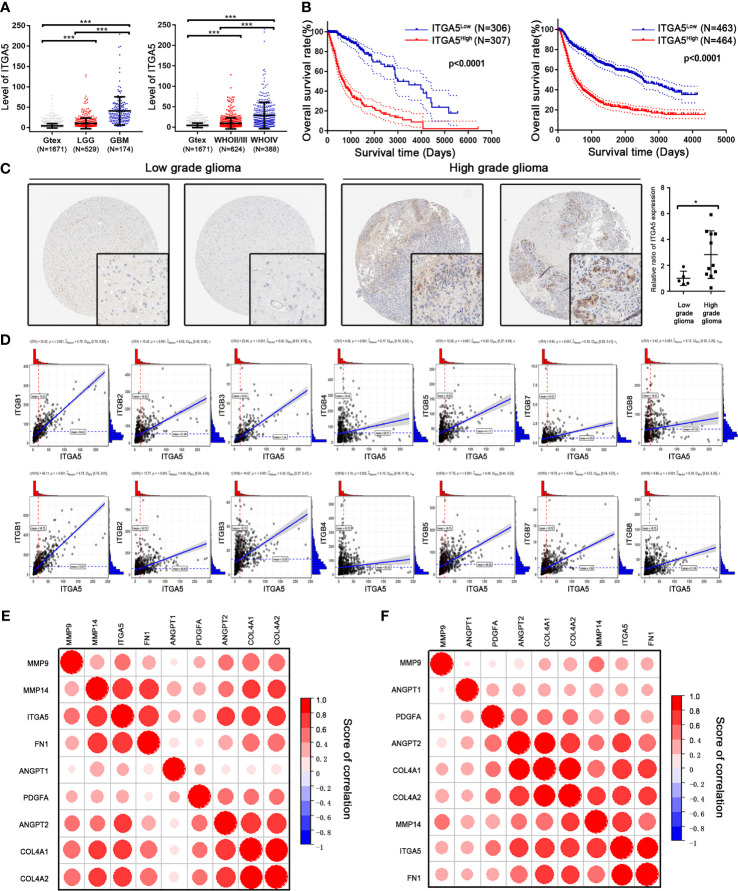
Identification of the prognostic value of *ITGA5* in glioma patients from The Cancer Genome Atlas (TCGA) and Chinese Glioma Genome Atlas (CGGA) databases. **(A)** Transcriptional levels of *ITGA5* in glioma patients from TCGA and CGGA. **(B)** Kaplan–Meier survival curves in glioma patients with high or low expressions of *ITGA5*. **(C)** Comparison of the protein levels of *ITGA5* in patients’ tissues with low- or high-grade glioma from the Human Protein Atlas (*p < 0.05; ***p < 0.01). **(D)** Correlation between *ITGA5* and different β-subunits of integrin using transcriptomic data from TCGA and CGGA. **(E, F)** Correlation between *ITGA5* and the genes responsible for vascular mimicry (VM) using transcriptomic data from TCGA **(E)** and CGGA **(F)** databases.

To determine the possible contribution of *ITGA5* in drug resistance, we systematically analyzed the transcriptomic data of glioma patients from TCGA and CGGA databases and normal brain samples from the GTEx database. The expression level of *ITGA5* was significantly higher in glioma tissues compared to that in normal brain tissue and steadily increased with advancing glioma grade (**Figure 4A**). A higher expression level of *ITGA5* was associated with poorer outcomes in patients with glioma (TCGA: HR = 5.564, 95%CI = 4.203–7.367, *p* < 0.0001; CGGA: HR = 2.899, 95%CI = 2.433–3.454, *p* < 0.0001) (**Figure 4B**). Consistent with this, more abundant *ITGA5* protein was observed in high-level gliomas than in low-level gliomas based on the immunohistochemistry staining files from THPA database (**Figure 4C**). Considering that forming a heterodimer with α- and β-subunits is necessary for integrin protein function, we calculated the correlation scores between *ITGA5* and different β-subunits and found that the expression of ITGB1 was highly correlated with *ITGA5* (**Figure 4D**). In addition, we also found that *ITGA5* was significantly positively correlated with VM-related genes, especially with genes coding collagen and other ECM components (**Figures 4E, F** **)**, indicating that *ITGA5* may increase the degree of malignancy and drug resistance of glioma by affecting VM formation.

**Figure 5:**
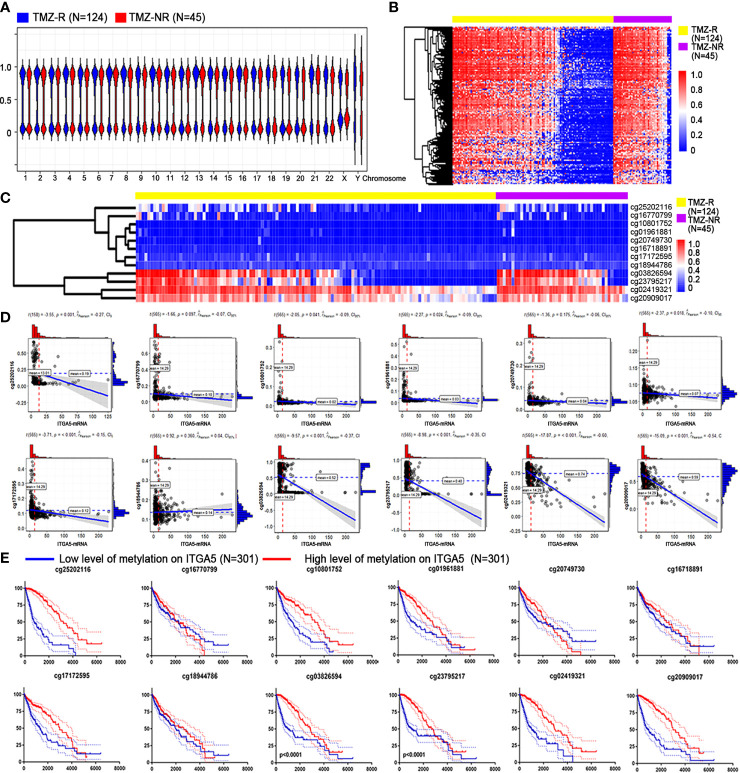
Association between *ITGA5* methylation with resistant properties in glioma patients. **(A)** Methylation landscape in temozolomide-resistant (TMZ-R) and non-resistant (TMZ-NR) glioma patients from The Cancer Genome Atlas (TCGA) database. **(B)** Differentially methylated sites associated with TMZ resistance in glioma patients. **(C)** Methylation levels of different sites on the *ITGA5* gene. **(D)** Correlation of the transcriptional level of *ITGA5* and the methylation level on its different sites. **(E)** Kaplan–Meier survival curves in glioma patients grouped by methylation level on the different sites of *ITGA5*.

Since methylation is pivotal in controlling expression patterns, we explored the DNA methylated landscape of the above patients. As exhibited in **Figure 5A**, a wider range of methylated genes occurred in the TMZ-NR group, which may explain why more genes were silenced and downregulated. With the thresholds of |fold change| > 1.2 and FDR < 0.05, there were 454 sites with decreased methylation in the TMZ-R group and only one site with increased methylation (**Figure 5B**). As for the *ITGA5* gene, we measured 12 probes targeting different areas on *ITGA5* and observed reduced methylation on sites of cg03826594 and cg2379527 in the TMZ-R group, both targeted on the body region of *ITGA5* gene (**Figure 5C**). In addition, a negative correlation existed between the methylation scores of these two sites and the gene expression of *ITGA5* (Pearson’s correlation coefficients of −0.37 and −0.35, respectively) (**Figure 5D**). Moreover, the reduced methylation on *ITGA5* was associated with poorer outcomes in glioma patients, reinforced by the survival analysis (**Figure 5E**). The above results indicated a striking correlation between reduced methylation and overexpression of *ITGA5* in drug-resistant glioma patients.

**Figure 6:**
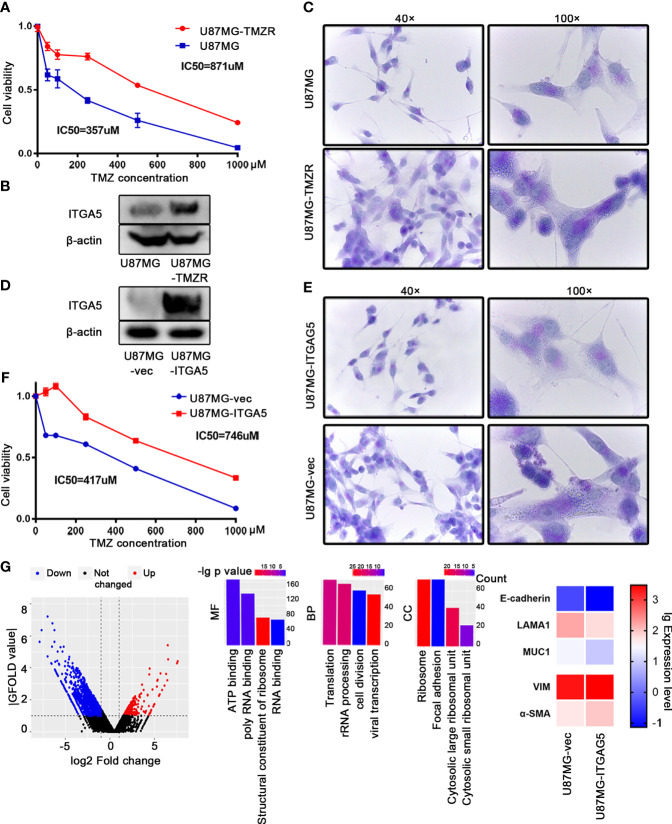
*ITGA5* promoted vascular mimicry (VM) formation and temozolomide (TMZ) resistance in glioma cells. **(A)** Confirmation of a TMZ-resistant (TMZ-R) cell line established using U87MG by Cell Counting Kit 8 (CCK8). **(B)** Examination of the expression of *ITGA5* in U87MG and U87MG-TMZR cells by Western blot. **(C)** Examination of VM in U87MG and U87MG-TMZR cells by periodic acid–Schiff (PAS) staining. **(D)** Measurement of *ITGA5* expression in *ITGA5*-overexpressing cells by Western blot. **(E)** Observation of VM in *ITGA5*-overexpressing cells by PAS staining. **(F)** Detection of tolerance to TMZ in *ITGA5*-overexpressing cells by CCK8. **(G)** Differentially expressed genes and pathways in *ITGA5*-overexpressing cells compared to control.

To confirm the role of *ITGA5* in drug resistance, we constructed the TMZ-resistant cell line named U87MG-TMZR, which exhibited a significantly increased resistance to TMZ as verified by the Cell Counting Kit 8 (CCK8) assay (**Figure 6A**). The results of Western blot showed augmented expression of *ITGA5* in TMZR cells compared to that in their parental cells, followed by stronger PAS staining (**Figures 6B, C** **)**. Then, we constructed and purified *ITGA5*-overexpressing cells, which was validated by Western blot (**Figure 6D**). *ITGA5*-overexpressing cells showed higher resistance to TMZ and a more enriched VM (**Figures 6E, F** **)**. Moreover, we performed transcriptomic sequencing using these two cell lines. The results showed 619 upregulated genes and 1,537 downregulated genes (|log2 fold change| > 1, |GFOLD| > 1). These DEGs were enriched in pathways related to transcription, translation, and cell division (**Figure 6G**), suggesting that *ITGA5* overexpression altered the cell proliferation activity. In addition, the epithelial features were decreased with increased mesenchymal genes in *ITGA5*-overexpressing cells, suggesting that *ITGA5* promoted the EMT process in glioma cells.

## Discussion

TMZ-based treatment remains a novel therapeutic approach in glioma patients; nonetheless, its therapeutic efficacy remains limited (33). Previous research works have demonstrated that TMZ pressed glioma cells to initiate the EMT process. As a result, TMZ-resistant cells have acquired an EMT-like phenomenon, represented by visible morphological changes (34). Likewise, the change of the morphology and polarity of glioma cells post-therapy changed the vascular environment, making it tolerant to anti-angiotherapy. Numerous clinical pieces of evidence suggest that EMT promotes VM formation, which is the leading cause of resistance to anti-angiotherapy targeting VEGF in glioma. On the one hand, glioma is a typical vascular-enriched solid tumor with highly heterogeneous vessel structure and sources (35). The rapid tumorigenesis of glioma makes it highly aerobic, especially in the central necrosis region (36). These areas disconnected from the endothelial tissue lack endothelium-dependent vessels, whereas VM channels are often observed nearby (37). Breakage of the existing vessels by chemicals or surgery forces the more robust formation of an intratumor blood supply system, to supply nutrients and oxygen and to remove waste products (38). A more remarkable VM appears in recurrent glioma, which may be induced by chemotherapy-induced hypoxia, to drive tumor resistance and relapse (39). On the other hand, chemical stress endows glioma cells with stemness; a fraction of these GBM stem-like cells can transdifferentiate into vascular smooth muscle-like cells to form VM (40). Furthermore, the signal to induce VM can be strengthened under Bevacizumab treatment in an IL-8/CXCR2-dependent pattern (41). Moreover, VM showed a delayed, but longer-lasting, vasculogenic activity, contributing to the failure of depriving tumors their blood supply (42). When endothelial-derived blood vessels are pharmacologically inhibited, the existence of VM can still support oxygen supply, greatly limiting the therapeutic effect of traditional anti-VEGF drugs (43).

At the beginning of this study, through investigating the DEGs and biological features between TMZ-R and TMZ-NR glioma patients, characteristics related to VM were focused on. In particular, U87MG-TMZR cells exhibited thicker invadopodia than did parental U87MG cells. Invadopodia are actin-rich structures that protrude from the plasma membrane; more robust invadopodia reflect more invasive properties and EMT activity. During radiotherapy or TMZ treatment in GBM cell lines, invadopodia increased along with ECM degradation and remodeling (44). Consistent with previous research, we also noticed that a much stronger activity of invadopodia presented in TMZ-R cells, revealing that the activity of invadopodia bridges the link between EMT and TMZ resistance in gliomas (45). The proteins and signaling pathways parallel in EMT and the generation of invadopodia support a strong association between them (46). The data presented here showed that *ITGA5* was greatly involved in these two cellular pathways. Since VM is accompanied by HIF-1α, *ITGA5* can also be directly induced by HIF-1α (47). *ITGA5* was observed to be localized in invadopodia, while its knockdown reduced the formation of invadopodia in U87MG cells (48).

Besides with *ITGA5*, the formation of VM is under the control of multiple ECM components. The major steps in VM formation include: the acquisition of stem-like ability to initiate cell differentiation, ECM remolding to form vascular-like morphology, and physiological connection between the VM channels and endothelial-lined vasculature. The family of integrins is greatly involved in cell adhesion and signal transduction between tumor cells and the ECM. *ITGA5* has also been verified to be located on circulating angiogenic cells in GBM, which affects neovascularization (27). After binding with its ligand, signals are transmitted to regulate FAK, PI3K, and other pathways, affecting tumor growth, invasion, and metastasis (49). Through identifying the “arginine (R)–glycine (G)–aspartate (D)” tripeptide sequence (RGD), integrin combines with ECM skeleton components such as fibrinogen 1 (FN1) and collagen 4 type IV (COL4A1 and COL4A2) to form a cross network in order to maintain the stability of the vascular microstructure (50). This step is assisted by the matrix metalloproteinases (MMP) family by targeting ECM proteins for degradation and releasing related growth factors. Platelet-derived growth factor (PDGF) and angiogenins (ANGPT1 and ANGPT2) can stabilize new blood vessels, while hepatic ligand receptor (EPHA2) signal transduction can regulate cell migration and intercellular adhesion, leading to the maturation of new blood vessels (51). As shown in **Figure 4**, the expression of *ITGA5* is positively correlated with the above ECM factors.

Previous literature has unearthed the existence of multiple DNA repair enzymes contributing to repair TMZ lesions and destroying its antitumor function, which also influenced *ITGA5* activity somehow. These mismatch repair (MMR) elements caused genetic alterations during the adaptation to TMZ, endowing glioma cells with new phenomenon in metabolism, proliferation, and immunogenicity. Thereinto, MGMT promoter methylation greatly impacts on MGMT protein expression and TMZ resistance in GBM (52). As exhibited in this article, the DNA methylation level was decreased genome-wide in the TMZ-R group, enabling recovery of the expressions of multiple genes compared to the non-resistant group. Of note is that the methylation level of *ITGA5* on two distinct sites (cg03826594 and cg2379527) also showed a close association with *ITGA5* expression and TMZ tolerance.

Considering that *ITAG5* is a promising therapeutic target, several drugs are undergoing clinical validation. Volociximab, a chimeric immunoglobulin gamma-4 (IgG4) monoclonal antibody binding to ITGA5/B1, demonstrated a favorable phase I safety profile as a single agent (53). MINT1526A (RG-7594) is another fully humanized, high-affinity, and function-blocking anti-human ITGA5/B1 IgG1 antibody. MINT1526A inhibits the binding of ITGA5/B1 with FN, thereby blocking ITGA5/B1-mediated endothelial cell adhesion, migration, and sprouting in matrices containing FN. It has also shown good tolerance and preliminary evidence of efficacy when combined with Bevacizumab (54). Cilengitide is an effective inhibitor of ITGAV/B3 and ITGAV/B5. Treating rats with a combination of bevacizumab and cilengitide significantly restricted tumor invasion than with bevacizumab only, suggesting that inhibiting *ITGA5* could replenish the antitumor ability of anti-VEGF agents (55).

In summary, *ITGA5*-induced VM may promote resistance to TMZ and Bevacizumab by altering glioma vascularization. Besides with briefing the origin and structural characteristics of VM, the elucidation of this mechanism is urgently needed for improving the outcomes of glioma patients and optimizing and developing individualized treatment strategies.

## Data Availability Statement

The datasets presented in this study can be found in online repositories. The names of the repository/repositories and accession number(s) can be found in the article/supplementary material.

## Author Contributions

YS, LC, and CX contributed to planning the study. MW, YL, LH, and LX performed the data collection and analysis. YS, HW, and ZL were responsible for the data interpretation. YS and CX drafted the manuscript. AW and LC revised the paper. All authors contributed to the article and approved the submitted version.

## Funding

This study was supported by the National Natural Science Foundation of China (no. 81873048) and Sichuan Provincial Science Fund for Distinguished Young Scholars of China (no. 2020JDJQ0065) to CX; the National Natural Science Foundation of China (General Program: no. 81672824; Key Program: no. U20A20380) to LC; Sichuan Science and Technology Program (no. 2021YFH0187) to YS; and the Fundamental Research Funds for the Central Universities (no. ZYGX2020KYQD002) to YS.

## Conflict of Interest

The authors declare that the research was conducted in the absence of any commercial or financial relationships that could be construed as a potential conflict of interest.

## Publisher’s Note

All claims expressed in this article are solely those of the authors and do not necessarily represent those of their affiliated organizations, or those of the publisher, the editors and the reviewers. Any product that may be evaluated in this article, or claim that may be made by its manufacturer, is not guaranteed or endorsed by the publisher.
